# Impact of the COVID-19 pandemic on psychosocial work factors and emotional exhaustion among workers in the healthcare sector: a longitudinal study among 1915 Dutch workers

**DOI:** 10.1136/oemed-2022-108478

**Published:** 2022-11-24

**Authors:** Fleur van Elk, Suzan J W Robroek, Alex Burdorf, Karen M Oude Hengel

**Affiliations:** 1 Department of Public Health, Erasmus University Medical Center, Rotterdam, The Netherlands; 2 Department of Work Health Technology, TNO, Leiden, The Netherlands

**Keywords:** COVID-19, health personnel, burnout, psychological, occupational health, longitudinal studies

## Abstract

**Objectives:**

This study aims to investigate across subgroups of healthcare workers (1) the changes in psychosocial working conditions and emotional exhaustion during the pandemic compared with the situation before, and (2) the impact of different stages of the COVID-19 pandemic in terms of hospital pressure on psychosocial working conditions and emotional exhaustion.

**Methods:**

Five questionnaire measurements during 2 years from 1915 healthcare workers in the longitudinal study ‘the Netherlands Working Conditions Survey-COVID-19’ were used. At each measurement, three subgroups were defined: working with patients with COVID-19, working with other patients and not working with patients. For each measurement, hospital pressure was determined by number of hospitalisations per day. Linear mixed models were fitted to analyse differences across subgroups of healthcare workers.

**Results:**

During COVID-19, psychosocial working conditions deteriorated among healthcare workers working with patients, in particular with patients with COVID-19, compared with healthcare workers not working with patients after correcting for the situation before COVID-19. No changes were observed for emotional exhaustion in any of the subgroups. An increasing hospital pressure improved job autonomy and reduced emotional demands among healthcare workers in COVID-19 wards, but had no influence on other psychosocial working conditions and emotional exhaustion.

**Conclusion:**

Psychosocial working conditions deteriorated for healthcare workers working with (COVID-19) patients during the pandemic, while emotional exhaustion did not change among all groups of healthcare workers.

**WHAT IS ALREADY KNOWN ON THIS TOPIC**

- There is a high prevalence of mental health problems among healthcare workers, before and during the COVID-19 pandemic.
- No consistent evidence exists on mental health among different types of healthcare workers during the COVID-19 pandemic.
- Unfavourable working conditions contribute to poor mental health outcomes among healthcare workers.

**WHAT THIS STUDY ADDS**

- During the COVID-19 pandemic, healthcare workers working with patients with COVID-19 or other patients experienced more unfavourable psychosocial working conditions compared with healthcare workers not working with patients.
- After adjustment for the pre-COVID-19 period, healthcare workers working with patients with COVID-19 or other patients experienced a larger deterioration in psychosocial working conditions compared with healthcare workers not working with patients, but no changes were observed for emotional exhaustion.
- An increasing hospital pressure due to more hospitalisations of patients with COVID-19 resulted in an improvement of job autonomy and emotional demands among healthcare workers in COVID-19 wards compared with healthcare workers not working with patients. It was not associated with other psychosocial working conditions and emotional exhaustion.

**HOW THIS STUDY MIGHT AFFECT RESEARCH, PRACTICE OR POLICY**

- Unfavourable psychosocial working conditions were already apparent among healthcare workers before the COVID-19 pandemic, and these conditions deteriorated during the pandemic. No effects were found for emotional exhaustion. Policy should focus on developing and implementing effective interventions to improve psychosocial working conditions of healthcare workers.

## Introduction

Healthcare workers are considered as a high-risk group to acquire COVID-19,^1 2^ or to die due to COVID-19,^3^ and they are at high risk to develop mental health problems due to high workload and time pressure. Recent systematic reviews have reported a variety of mental health problems among healthcare workers during COVID-19, such as anxiety, depression, stress and post-traumatic stress symptoms,^4–9^ psychological distress,^10^ insomnia,^8 11^ burnout and burnout-related outcomes such as emotional exhaustion, depersonalisation and low personal accomplishment.^12 13^ These systematic reviews included studies mostly focusing on doctors and nurses, but also midwives, allied healthcare workers, pharmacists and technicians. A high prevalence of mental health problems was already apparent among healthcare workers before the COVID-19 pandemic.^14^ For example, paediatric, emergency and primary care nurses had a high prevalence of burnout and showed moderate to high levels of emotional exhaustion (28%–41%), depersonalisation (15%–44%) and a lack of personal accomplishment (31%–43%).^15–17^ To date, only cross-sectional research studied the impact of working with patients with COVID-19 or not on mental health. While some studies showed higher levels of mental health problems (such as burnout, secondary trauma, anxiety and depression) among healthcare workers working with patients with COVID-19,^18–20^ other studies showed that non-frontline healthcare workers had higher levels of mental health problems (such as depression, anxiety and resilience).^21 22^

Unfavourable working conditions may contribute to poor mental health among healthcare workers.^14^ Recent studies have shown that the COVID-19 pandemic was associated with adverse psychosocial working conditions, such as high emotional work demands and experiencing a high workload,^23^ especially among healthcare workers working with patients with COVID-19.^24^ Two recent studies among healthcare workers compared working conditions and recovery during COVID-19 with pre-COVID-19. These studies found that compared with prepandemic, healthcare workers had poorer working conditions and worse recovery,^25 26^ and that healthcare workers working with patients with COVID-19 reported poorer working conditions than other healthcare workers.^25^ It is, however, a limitation that participants were not followed up at the individual level.

Based on the job demands-resources (JD-R) model, working conditions can be categorised into job demands (eg, physical workload, time pressure, environment, shift work) and job resources (eg, feedback, rewards, job control, supervisor support). An imbalance between demands and resources could result in job strain and therefore occupational stress. The JD-R model provides strong evidence that job demands are primarily related to the emotional exhaustion component of burnout.^27^

The current study will contribute to the existing literature in three ways. First, the current literature among healthcare workers lacks longitudinal studies comparing the effects of the COVID-19 pandemic on changes in psychosocial working conditions and mental health before and during the pandemic at the individual level. As unfavourable working conditions and mental health problems were already present among healthcare workers before the COVID-19 pandemic, it is important to compare the associations of unfavourable working conditions and mental health problems during the pandemic with the pre-COVID-19 situation. Second, the COVID-19 pandemic is not a fixed stage, meaning that infection rates, hospitalisations and governmental measures differed over time. Therefore, it is relevant to gain insight in the effects of different stages of the COVID-19 pandemic on psychosocial working conditions and mental health. Thereby, differentiation across groups of healthcare workers is needed as hospital pressure is higher for COVID-19 wards than other departments. The aim of the current study is to investigate (1) changes in psychosocial working conditions and emotional exhaustion during the pandemic compared with the situation before across subgroups of healthcare workers, and (2) the impact of different stages of the COVID-19 pandemic in terms of hospital pressure on psychosocial working conditions and emotional exhaustion across these subgroups.

## Methods

### Data

The current study is embedded within the longitudinal study ‘the Netherlands Working Conditions Survey COVID-19’ (NWCS-COVID-19), which is a cohort study that consists of one measurement before (baseline measurement) and four measurements during the COVID-19 pandemic.^28^ The baseline measurement took place in November 2019. The second measurement was in July 2020, shortly after the first COVID-19 wave when governmental measures were relaxed (eg, opening of all sectors, primary schools and day care). During the period of this second measurement, the infection rates and hospitalisations (average of 127 per day across the Netherlands) were low. The third measurement was in November 2020, at the start of the second COVID-19 wave when restaurants, bars and the entertainment sector were closed, and non-essential shops had restricted opening hours. During this month, the infection rates were moderately high, and the average total hospitalisations per day was 2032. The fourth measurement was in March 2021 when the government set further restrictions, such as the evening curfew and the obligation to visit non-essential shops by appointment only, and vaccinations started among healthcare workers. During this month, the infection rates were quite similar to the third measurement, and hospitalisations were on average 2051 per day. The fifth measurement was in November 2021 right before the Omicron wave when governmental measures were restricted after a period of relaxation, and vaccination rates were high among healthcare workers but booster vaccination had not started yet. During the period of this fifth measurement the infection rates were again moderately high, and hospitalisations were on average 2310 per day.

### Study population

**Figure 1:**
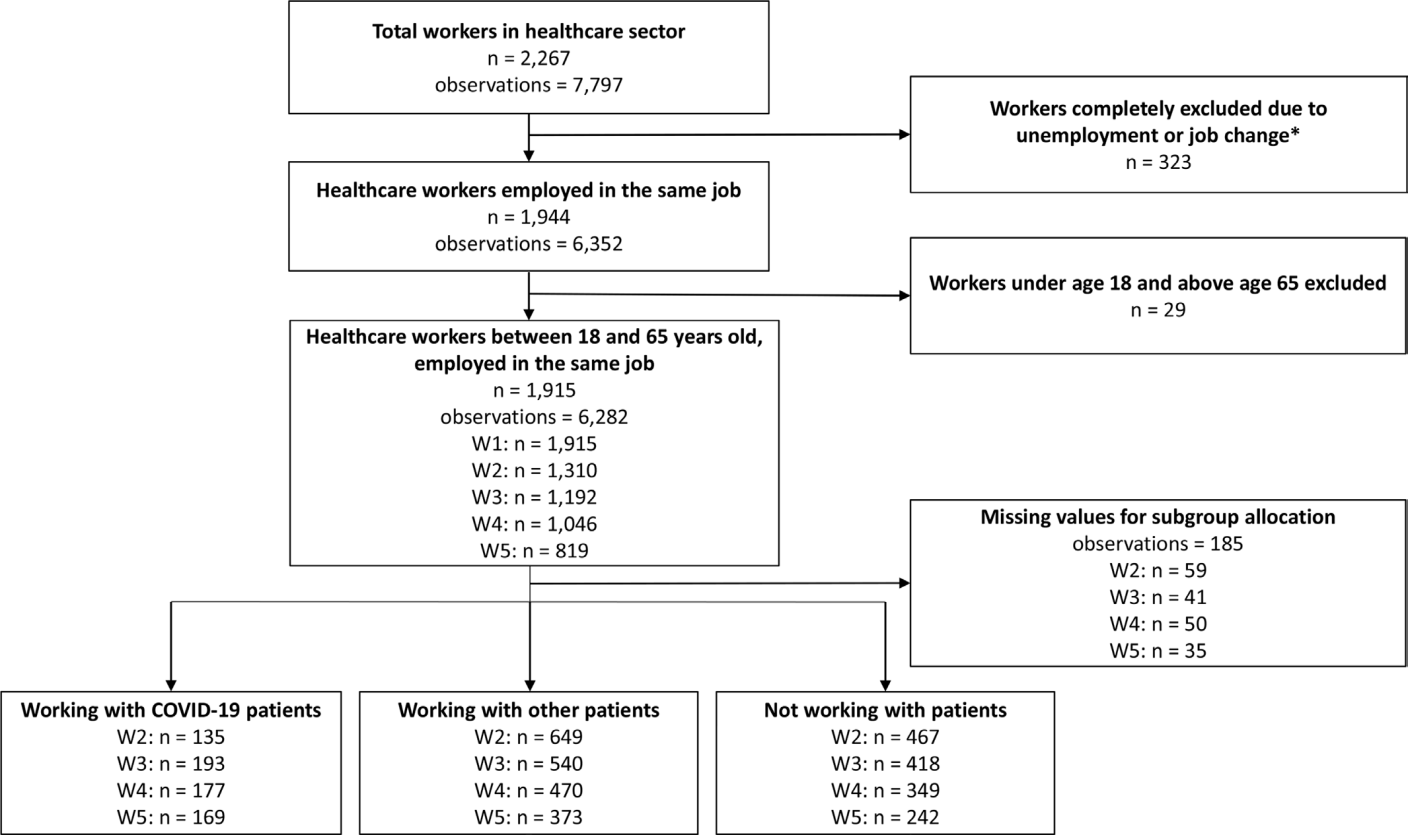
Flow chart of the selection procedure of the study population. W1=measurement wave 1 (November 2019), W2=measurement wave 2 (June 2020, hospital pressure 0.04), W3=measurement wave 3 (November 2020, hospital pressure 0.53), W4=measurement wave 4 (March 2021, hospital pressure 0.46), W5=measurement wave 5 (November 2021, hospital pressure 0.53). *When an observation was excluded because of unemployment or job change, the subsequent observations were also excluded. When all observations after baseline were excluded, the worker was excluded completely.

The target population of this study were Dutch workers in the healthcare sector between the ages of 18 and 65 years who participated in the baseline measurement and at least one follow-up measurement. The study population consists of healthcare workers working—among others—in hospitals, nursing homes and in home care. When workers became unemployed or changed their job at (one of) the follow-up measurements, this observation and the following observations were excluded. The final analytical sample included 6282 observations from 1915 respondents (figure 1).

Based on questions about whether the healthcare workers worked with patients and if they did, whether they worked with patients with COVID-19, healthcare workers were divided into three subgroups: (1) working with patients with COVID-19, (2) working with other patients and (3) not working with patients. Since workers could change their group status over time, the number of workers per group is not fixed across the measurements (figure 1).

### Outcome measures

#### Emotional exhaustion

Emotional exhaustion was measured with the emotional exhaustion scale of the validated Utrecht Burnout Scale.^29^ This scale consists of five items such as ‘I feel emotionally exhausted by my work’ and ‘At the end of a working day, I feel empty’. The items can be answered on a 7-point scale ranging from ‘never’ to ‘every day’. The average score (ranging from 1 to 7) of these five items was used as continuous measure for emotional exhaustion.

#### Psychosocial working conditions

##### Job autonomy

Job autonomy was based on five items of the Job Content Questionnaire (JCQ) with questions on decision-making at the job, deciding on the order and speed of conducting tasks, having to find solutions for problems and being able to take time off from work.^30^ The items were answered with ‘yes, regularly’, ‘yes, sometimes’ or ‘no’. The average score (ranging from 1 to 3) of these five items was used as continuous measure for job autonomy. A higher score means more job autonomy.

##### Psychological job demands

Another three items of the JCQ were used to measure psychological job demands. The items assess the speed, amount of work and difficulty level of the job,^30^ and were answered on a 4-point scale ranging from ‘never’ to ‘always’. The average score (ranging from 1 to 4) of these three items was used as continuous measure for psychological job demands. A higher score means more psychological job demands.

##### Emotional demands

To measure emotional demands of the job, three items of the Copenhagen Psychosocial Questionnaire were used.^31^ These items concern questions about emotionally difficult situations, emotional demands and emotional involvement with the job. The items were answered on a 4-point scale ranging from ‘never’ to ‘always’. The average score (ranging from 1 to 4) of these three items was used as continuous measure for emotional demands. A higher score means more emotional demands.

##### Social support from colleagues

Social support from colleagues was based on two items of the JCQ.^30^ These items concern personal interest and friendliness of colleagues, and were answered on a 4-point scale ranging from ‘totally disagree’ to ‘totally agree’. The average score (ranging from 1 to 4) of these two items was used as continuous measure for social support from colleagues, and a higher score means more social support.

##### Social support from the supervisor

Social support from the supervisor was based on two items of the JCQ.^30^ These items concern interest of the supervisor in well-being of the worker and paying attention when talking to the worker, which were answered on a 4-point scale ranging from ‘totally disagree’ to ‘totally agree’. The average score (ranging from 1 to 4) of these two items was used as continuous measure for social support from the supervisor, and a higher score means more social support.

The average scores of all outcome measures were used as continuous variables, because no clear cut-offs exist.

### Hospital pressure

Hospital pressure was determined as a daily number of patients with COVID-19 hospitalised in the Netherlands.^32^ For each day during the measurement period, the number of hospitalisations was derived. The daily average of these numbers across the time window of collecting data was calculated, taking into account the amount of respondents at each day. Based on the average number of hospitalisations during the measurement period, we calculated a score from 0 to 1, where 1 is the peak number of hospitalisations during the entire pandemic (4322 hospitalisations in March 2020). This resulted in the following values for hospital pressure: 0.04 (measurement 2), 0.53 (measurement 3), 0.46 (measurement 4) and 0.53 (measurement 5).

### Covariates

Age, sex, household composition, level of education and working hours at baseline were included as covariates. Age was divided into four categories, namely 19–34, 35–44, 45–54 and 55–64. Gender was a dichotomous variable (male/female). Household composition was categorised into: single (with or without children), having a partner and no children, or having a partner and children. Level of education was divided into three categories: low, intermediate or high. The number of working hours at baseline was included as continuous variable.

### Statistical analysis

Linear mixed models were fitted for the five psychosocial working conditions and emotional exhaustion as dependent variables. The models contained random intercepts for the within-person variation. Time-dependent fixed effects were hospital pressure and subgroup status. Time-independent fixed effects were sex, age, household composition, level of education and working hours. First, differences over time across subgroups were estimated, adjusted for individual characteristics with adjustments for baseline scores of the dependent variables. Second, the impact of different stages of the COVID-19 pandemic across subgroups was estimated using interaction terms between subgroups and hospital pressure. Hospital pressure was used as time-varying variable since the aim of the study is to measure the impact of different stages of the COVID-19 pandemic. Time of measurement was not included in the models to prevent overadjustment. All linear mixed model analyses were also performed with stratification by sex. For descriptive purposes, the differences across subgroups were estimated without adjusting for baseline scores of the dependent variables. Additionally, fixed effects analyses were performed to investigate the effect of a change from working with other patients to working with patients with COVID-19 on the change in psychosocial working conditions and emotional exhaustion. All analyses were performed using IBM SPSS software V.28 with the unstructured repeated covariance type and the restricted maximum likelihood method in the linear mixed models procedure. The supplementary fixed effects models were performed as linear regression analyses, both with and without adjusting for hospital pressure. Hedge’s d effect sizes, expressing the observed difference relative to the pooled SD, were calculated to compare the three groups of healthcare workers for differences in psychosocial working conditions and exhaustion. Logistic regression analyses were used to evaluate non-response bias due to selective dropout during the follow-up measurements.

## Results

Regarding the non-response analyses, respondents in measurement 3 had a higher job autonomy and more social support from the supervisor at measurement 2 than those who dropped out after measurement 2 (online supplemental table 1). No differences were found for other measurements. The study population consisted of 1915 workers in the healthcare sector (table 1). Across all measurements, the majority was female (81%–82%), with 32% aged 55–65 years at baseline. The proportion of healthcare workers working with patients with COVID-19 increased from 10% in measurement 2, 16% in measurement 3 and 17% in measurement 4 to 21% in measurement 5. In conjunction with this trend, the proportion of healthcare workers working with other patients or not working with patients decreased slightly over time (table 1). The psychosocial working conditions and emotional exhaustion scores are not to moderately correlated with each other, with correlation coefficients lower than 0.4.^33^

10.1136/oemed-2022-108478.supp1 Supplementary data

**Table 1:** Characteristics of healthcare workers in the Netherlands (n=1915 at baseline) by period of measurement

	Measurement 1 November 2019 (n=1915)	Measurement 2 July 2020 (n=1310)	Measurement 3 November 2020 (n=1192)	Measurement 4 March 2021 (n=1046)	Measurement 5 November 2021 (n=819)
Age (%)					
19–34	506 (26)	325 (25)	284 (24)	224 (21)	150 (18)
35–44	356 (19)	237 (18)	207 (17)	184 (18)	150 (18)
45–54	438 (23)	303 (23)	286 (24)	256 (25)	222 (27)
55–65	615 (32)	445 (34)	415 (35)	382 (36)	297 (37)
Sex (% female)	1563 (82)	1061 (81)	970 (81)	861 (82)	671 (82)
Household composition (%)					
Single	430 (23)	288 (22)	275 (23)	249 (24)	185 (22)
Having a partner	638 (33)	428 (33)	418 (35)	355 (34)	275 (34)
Having a partner with child(ren)	847 (44)	594 (45)	499 (42)	442 (42)	359 (44)
Level of education (%)					
Low	110 (6)	80 (6)	69 (6)	67 (6)	48 (6)
Middle	765 (40)	522 (40)	468 (39)	419 (40)	332 (41)
High	1036 (54)	706 (54)	652 (55)	560 (54)	437 (53)
Working hours per week (mean, SD)*	26.90 (8.99)	–	18.80 (7.36)	28.52 (7.27)	28.26 (7.66)
Subgroup (%)					
Working with patients with COVID-19	–	135 (10)	193 (16)	177 (17)	169 (21)
Working with other patients	–	649 (50)	540 (45)	470 (45)	373 (46)
Not working with patients	–	467 (35)	418 (35)	349 (33)	342 (30)
Working conditions (mean, SD)					
Job autonomy (1–3)	2.40 (0.49)	2.33 (0.52)	2.34 (0.52)	2.33 (0.53)	2.33 (0.52)
Psychological job demands (1–4)	2.44 (0.63)	2.34 (0.58)	2.35 (0.58)	2.31 (0.58)	2.39 (0.64)
Emotional demands (1–4)	2.21 (0.55)	2.20 (0.53)	2.18 (0.54)	2.14 (0.51)	2.17 (0.55)
Social support from colleagues (1–4)	3.43 (0.56)	3.47 (0.55)	3.48 (0.54)	3.48 (0.52)	3.48 (0.54)
Social support from supervisor (1–4)	3.01 (0.66)	3.08 (0.67)	3.08 (0.67)	3.11 (0.64)	3.07 (0.67)
Emotional exhaustion (1–7) (mean, SD)	2.34 (1.25)	2.42 (1.24)	2.40 (1.24)	2.35 (1.25)	2.51 (1.37)

*Not available for measurement 2.

Table 2 shows the results, after adjustment for the baseline scores, in psychosocial working conditions and emotional exhaustion during COVID-19 compared with pre-COVID-19. A larger decrease in job autonomy and social support from the supervisor, and a larger increase in psychological job demands and emotional demands were found for both workers working with patients with COVID-19 and other patients compared with workers not working with patients. A larger increase in social support from colleagues was only found for healthcare workers working in COVID-19 wards. No statistically significant differences were found across subgroups in changes in emotional exhaustion. Effect sizes comparing healthcare workers working with patients with COVID-19 to healthcare workers working without patients ranged from 0.08 (social support from supervisor) to 0.20 (job autonomy). The effect sizes were lower when comparing healthcare workers in non-COVID-19 wards to healthcare workers not working with patients (online supplemental table 2).

**Table 2:** Linear mixed model estimates for differences across healthcare workers in changes in psychosocial working conditions and emotional exhaustion during the COVID-19 pandemic, compared with baseline scores

	Job autonomy (1–3)	Psychological job demands (1–4)	Emotional demands (1–4)	Social support from colleagues (1–4)	Social support from supervisor (1–4)	Emotional exhaustion (1–7)
	β (95% CI)	β (95% CI)	β (95% CI)	β (95% CI)	β (95% CI)	β (95% CI)
Subgroup						
Working with patients with COVID-19	−**0.12 (−0.15 to** −**0.08)**	**0.07 (0.03 to 0.12)**	**0.11 (0.07 to 0.15)**	**0.08 (0.03 to 0.13)**	−**0.08 (−0.14 to** −**0.01)**	0.06 (−0.04 to 0.16)
Working with other patients	−**0.08 (−0.11 to** −**0.05)**	**0.04 (0.002 to 0.07)**	**0.08 (0.04 to 0.11)**	0.03 (−0.01 to 0.07)	−**0.07 (−0.12 to** −**0.02)**	0.04 (−0.04 to 0.11)
Not working with patients	Ref	Ref	Ref	Ref	Ref	Ref

Bold indicates statistical significance (p<0.05). Analyses were corrected for sex, age, household composition, level of education, working hours and baseline score of the specific working condition.

An increase in hospital pressure resulted in a larger increase in job autonomy and a larger decrease in emotional demands among healthcare workers in COVID-19 wards compared with healthcare workers not working with patients (table 3). Interaction effects were observed whereby higher numbers of patients with COVID-19 being hospitalised were associated with increased job autonomy and decreased emotional demands (table 3).

**Table 3:** Linear mixed model estimates for the influence of hospital pressure on differences across healthcare workers in changes in psychosocial working conditions and emotional exhaustion

	Job autonomy (1–3)	Psychological job demands (1–4)	Emotional demands (1–4)	Social support from colleagues (1–4)	Social support from supervisor (1–4)	Emotional exhaustion (1–7)
	β (95% CI)	β (95% CI)	β (95% CI)	β (95% CI)	β (95% CI)	β (95% CI)
Subgroup						
Working with patients with COVID-19	−**0.18 (−0.24 to** −**0.11)**	0.07 (−0.01 to 0.15)	**0.20 (0.12 to 0.28)**	0.08 (−0.01 to 0.17)	−**0.14 (−0.26 to** −**0.03)**	−0.06 (−0.23 to 0.11)
Working with other patients	−**0.08 (−0.12 to** −**0.04)**	0.04 (−0.01 to 0.09)	**0.08 (0.03 to 0.13)**	0.04 (−0.02 to 0.10)	−**0.09 (−0.16 to** −**0.02)**	−0.02 (−0.12 to 0.09)
Not working with patients	Ref	Ref	Ref	Ref	Ref	Ref
Hospital pressure	−0.03 (−0.10 to 0.04)	0.01 (−0.07 to 0.10)	−0.02 (−0.10 to 0.06)	0.01 (−0.09 to 0.11)	−0.05 (−0.17 to 0.07)	−0.10 (−0.27 to 0.07)
Interaction between subgroup and hospital pressure						
Working with patients with COVID-19 × hospital pressure	**0.15 (0.02 to 0.29)**	0.01 (−0.17 to 0.18)	−**0.22 (−0.38 to** −**0.06)**	−0.01 (−0.21 to 0.20)	0.18 (−0.07 to 0.43)	0.32 (−0.03 to 0.67)
Working with other patients × hospital pressure	0.01 (−0.08 to 0.10)	−0.0003 (−0.11 to 0.11)	−0.02 (−0.12 to 0.09)	−0.03 (−0.17 to 0.10)	0.06 (−0.10 to 0.22)	0.17 (−0.06 to 0.40)
Not working with patients × hospital pressure	Ref	Ref	Ref	Ref	Ref	Ref

Bold indicates statistical significance (p<0.05). Analyses were corrected for sex, age, household composition, level of education, working hours and baseline score of the specific working condition.

Regarding the effects for sex, no or small differences were found between males and females (see online supplemental table 3 for the descriptives of the study population, stratified by sex).

Online supplemental table 4 shows the differences across subgroups without adjusting for baseline scores of the dependent variables.

The fixed effects analyses within individuals (online supplemental table 5) show that starting work with patients with COVID-19 was associated with statistically significant increased emotional exhaustion and increased social support from colleagues. All other psychosocial working conditions did not change.

## Discussion

After correcting for the pre-COVID-19 pandemic situation, healthcare workers working with patients, in particular those working in COVID-19 wards, experienced more unfavourable psychosocial working conditions compared with other healthcare workers during the pandemic. The exception was social support from colleagues, which increased during the pandemic. However, all effect sizes are small, since a maximum effect size of 0.20 is considered a small effect. The effect sizes correspond with a maximum shift in job autonomy of 0.12 (4%) on a scale from 1 to 3. No differences in emotional exhaustion were found between subgroups of healthcare workers. Unexpectedly, an increase in hospital pressure resulted in improvements of job autonomy and emotional demands among healthcare workers in COVID-19 wards.

Our study is in line with a previous study that showed an association between the COVID-19 pandemic and adverse psychosocial working conditions such as high emotional demands and high workload.^23^ Other research also showed that the psychosocial workload is higher among healthcare workers in COVID-19 wards compared with other healthcare workers.^24^ Studies with repeated cross-sectional data from before and during COVID-19 already found that during COVID-19 working conditions were poorer compared with pre-COVID-19 among healthcare workers in COVID-19 wards,^25 26^ which is now supported by the current longitudinal study results. The change from working at a general patient ward to working with patients with COVID-19 during the pandemic did not change job autonomy, psychological job demands, emotional demands and social support from the supervisor. This indicates that the deterioration of these working conditions at that time did not depend on work activities. Social support from colleagues increased, indicating that the improvement in social support from colleagues resulted from working in COVID-19 wards. Regarding the different directions of social support from colleagues and supervisors, current literature lacks theories to explain this. However, a possible explanation could be a form of social sharing of emotions,^34^ namely more support among colleagues who experienced an equal situation and worked together to reach a bigger goal. On the contrary, supervisors might have had other responsibilities, such as arranging sufficient protective equipment and personnel, rearranging beds and implementing new measurements, at the expense of supporting personnel.

No differences in emotional exhaustion over time were found between subgroups of healthcare workers, even though the results show that healthcare workers working with patients, especially those working with patients with COVID-19, experienced adverse psychosocial working conditions. While the JD-R model states that more job demands lead to more emotional exhaustion,^27^ the effect sizes might be too small to expect a change in emotional exhaustion. The lack of effect can also be explained by the timing of the measurement. Our first measurement during the pandemic was directly after the first peak, in which the hospital pressure was relatively low. It could be hypothesised that stress and emotional exhaustion were higher during the first months of the pandemic due to many uncertainties, such as shortage of protective equipment and uncertainty about the protocols and treatment of COVID-19. While the other three measurements were conducted during one of the COVID-19 waves, they did not take place during the weeks with the highest hospitalisations, which might explain the lack of effect in emotional exhaustion across the subgroups. Another possibility is that burnout, of which emotional exhaustion is a dimension, would occur later on, since burnout symptoms usually become apparent after experiencing stress during a prolonged time. Moreover, previous longitudinal studies reported highest levels of emotional exhaustion among nurses compared with other healthcare workers during the pandemic.^35 36^ Therewith, it could be hypothesised that stratification by occupation could have shown different results across different groups. Unfortunately, this study lacked power to distinguish between occupations. The results are also in contrast with cross-sectional studies showing differences in mental health problems between frontline and non-frontline workers during COVID-19, such as burnout and stress.^18 19 21^ These contrary results could be explained by the differences in study design, timing of measurements, different type of healthcare workers and outcomes.

Surprisingly, an increase in hospital pressure resulted in a more favourable change in job autonomy and emotional demands among healthcare workers working with patients with COVID-19. A reason for the increase in autonomy could be that during higher hospital pressure, healthcare workers are expected to be more self-reliant due to the hectic and new situations at the workplace. An explanation for the decrease in emotional demands could be related to the JD-R model. Research shows that high social support at work may moderate the adverse impact of jobs with high job demands.^37^ The increase in collegial social support among healthcare workers who started to work with patients with COVID-19 could have had a positive effect on the relationship between hospital pressure and emotional demands. Further research is needed to study why healthcare workers, who are expected to be exposed most to the increase in hospital pressure, experienced an improvement in job autonomy and emotional demands.

### Strengths and limitations

A major strength of this study is the longitudinal data with measurements before and during COVID-19, which made it possible to study psychosocial working conditions and emotional exhaustion during the pandemic and compare them to the period before the pandemic in 2019. Another strength is the possibility to divide the healthcare workers into three different groups to further disentangle the impact of COVID-19 on psychosocial working conditions and emotional exhaustion.

However, the study also goes with limitations. First, only the respondents who gave permission in 2019 were contacted to participate in the NWCS-COVID-19 follow-up study, possibly leading to selection bias. Second, we considered a mediation analysis to further disentangle the relationship between psychosocial working conditions and emotional exhaustion. However, this was not feasible due to changes in subgroup status across the measurement periods. Third, the timing of the follow-up measurements was before or after a COVID-19 peak, which means during periods of less hospitalisations. This could have had an effect on the experienced psychosocial working conditions at the time the measurements took place, whereas the results could have been different when measured during peaks of hospitalisations. Fourth, only emotional exhaustion has been assessed as a measure of burnout. However, other factors could also be related to burnout, such as workplace justice, reward and job insecurity.^38^ Fifth, the results are based on healthcare workers, decreasing the external validity towards other occupations. At last, the power in the outcomes of table 3, including the interaction between subgroups and hospital pressure, is limited.

## Conclusion

Healthcare workers working with patients with COVID-19 experience the most unfavourable psychosocial working conditions, and these working conditions deteriorated during COVID-19 compared with the period before the pandemic. However, an increase in hospital pressure has no further deteriorating effects on psychosocial working conditions; job autonomy and emotional demands even improved. Unfavourable psychosocial working conditions were already apparent among healthcare workers before the COVID-19 pandemic, and the current results show that these conditions deteriorated during the pandemic. Policy should focus on developing and implementing effective interventions to improve the psychosocial working conditions of healthcare workers.

## Data Availability

Data are available upon reasonable request. Data are stored at TNO, Unit Healthy Living, the Netherlands. Data are available upon reasonable request by the last author.

## References

[1] Koh D . Occupational risks for COVID-19 infection. Occup Med 2020;70:3–5. 10.1093/occmed/kqaa036 PMC7107962 32107548

[2] van der Plaat DA , Madan I , Coggon D , et al . Risks of COVID-19 by occupation in NHS workers in England. Occup Environ Med 2022;79:176–83. 10.1136/oemed-2021-107628 34462304

[3] Nafilyan V , Pawelek P , Ayoubkhani D , et al . Occupation and COVID-19 mortality in England: a national linked data study of 14.3 million adults. Occup Environ Med 2022;79:433–41. 10.1136/oemed-2021-107818 34965981

[4] Saragih ID , Tonapa SI , Saragih IS , et al . Global prevalence of mental health problems among healthcare workers during the Covid-19 pandemic: a systematic review and meta-analysis. Int J Nurs Stud 2021;121:104002. 10.1016/j.ijnurstu.2021.104002 34271460 PMC9701545

[5] Li Y , Scherer N , Felix L , et al . Prevalence of depression, anxiety and post-traumatic stress disorder in health care workers during the COVID-19 pandemic: a systematic review and meta-analysis. PLoS One 2021;16:e0246454. 10.1371/journal.pone.0246454 33690641 PMC7946321

[6] Yan H , Ding Y , Guo W . Mental health of medical staff during the coronavirus disease 2019 pandemic: a systematic review and meta-analysis. Psychosom Med 2021;83:387–96. 10.1097/PSY.0000000000000922 33818054

[7] da Silva Neto RM , Benjamim CJR , de Medeiros Carvalho PM , et al . Psychological effects caused by the COVID-19 pandemic in health professionals: a systematic review with meta-analysis. Prog Neuropsychopharmacol Biol Psychiatry 2021;104:110062. 10.1016/j.pnpbp.2020.110062 32771337 PMC7409979

[8] Ghahramani S , Kasraei H , Hayati R , et al . Health care workers' mental health in the face of COVID-19: a systematic review and meta-analysis. Int J Psychiatry Clin Pract 2022:1–10. 10.1080/13651501.2022.2101927 35875844

[9] Xiong N , Fritzsche K , Pan Y , et al . The psychological impact of COVID-19 on Chinese healthcare workers: a systematic review and meta-analysis. Soc Psychiatry Psychiatr Epidemiol 2022;57:1515–29. 10.1007/s00127-022-02264-4 35325261 PMC8943357

[10] Sasaki N , Kuroda R , Tsuno K , et al . The deterioration of mental health among healthcare workers during the COVID-19 outbreak: a population-based cohort study of workers in Japan. Scand J Work Environ Health 2020;46:639–44. 10.5271/sjweh.3922 32905601 PMC7737801

[11] Pappa S , Ntella V , Giannakas T , et al . Prevalence of depression, anxiety, and insomnia among healthcare workers during the COVID-19 pandemic: a systematic review and meta-analysis. Brain Behav Immun 2020;88:901–7. 10.1016/j.bbi.2020.05.026 32437915 PMC7206431

[12] Gualano MR , Sinigaglia T , Lo Moro G , et al . The burden of burnout among healthcare professionals of intensive care units and emergency departments during the COVID-19 pandemic: a systematic review. Int J Environ Res Public Health 2021;18:8172. 10.3390/ijerph18158172 34360465 PMC8346023

[13] Galanis P , Vraka I , Fragkou D , et al . Nurses' burnout and associated risk factors during the COVID-19 pandemic: a systematic review and meta-analysis. J Adv Nurs 2021;77:3286–302. 10.1111/jan.14839 33764561 PMC8250618

[14] Weinberg A , Creed F . Stress and psychiatric disorder in healthcare professionals and hospital staff. Lancet 2000;355:533–7. 10.1016/S0140-6736(99)07366-3 10683003

[15] Pradas-Hernández L , Ariza T , Gómez-Urquiza JL , et al . Prevalence of burnout in paediatric nurses: a systematic review and meta-analysis. PLoS One 2018;13:e0195039. 10.1371/journal.pone.0195039 29694375 PMC5918642

[16] Li H , Cheng B , Zhu XP . Quantification of burnout in emergency nurses: a systematic review and meta-analysis. Int Emerg Nurs 2018;39:46–54. 10.1016/j.ienj.2017.12.005 29361420

[17] Monsalve-Reyes CS , San Luis-Costas C , Gómez-Urquiza JL , et al . Burnout syndrome and its prevalence in primary care nursing: a systematic review and meta-analysis. BMC Fam Pract 2018;19:59. 10.1186/s12875-018-0748-z 29747579 PMC5944132

[18] Trumello C , Bramanti SM , Ballarotto G , et al . Psychological adjustment of healthcare workers in Italy during the COVID-19 pandemic: differences in stress, anxiety, depression, burnout, secondary trauma, and compassion satisfaction between frontline and non-frontline professionals. Int J Environ Res Public Health 2020;17:8358. 10.3390/ijerph17228358 33198084 PMC7696387

[19] Lai J , Ma S , Wang Y , et al . Factors associated with mental health outcomes among health care workers exposed to coronavirus disease 2019. JAMA Netw Open 2020;3:e203976. 10.1001/jamanetworkopen.2020.3976 32202646 PMC7090843

[20] Li J , Su Q , Li X , et al . COVID-19 negatively impacts on psychological and somatic status in frontline nurses. J Affect Disord 2021;294:279–85. 10.1016/j.jad.2021.07.031 34304082 PMC8286238

[21] An Y , Sun Y , Liu Z , et al . Investigation of the mental health status of frontier-line and non-frontier-line medical staff during a stress period. J Affect Disord 2021;282:836–9. 10.1016/j.jad.2020.12.060 33601725 PMC7773008

[22] Baminiwatta A , De Silva S , Hapangama A , et al . Impact of COVID-19 on the mental health of frontline and non-frontline healthcare workers in Sri Lanka. Ceylon Med J 2021;66:16–31. 10.4038/cmj.v66i1.9351 34983177

[23] Giménez-Espert MDC , Prado-Gascó V , Soto-Rubio A . Psychosocial risks, work engagement, and job satisfaction of nurses during COVID-19 pandemic. Front Public Health 2020;8:566896. 10.3389/fpubh.2020.566896 33330313 PMC7716584

[24] Shoja E , Aghamohammadi V , Bazyar H , et al . Covid-19 effects on the workload of Iranian healthcare workers. BMC Public Health 2020;20:1636. 10.1186/s12889-020-09743-w 33138798 PMC7605333

[25] Jonsdottir IH , Degl'Innocenti A , Ahlstrom L , et al . A pre/post analysis of the impact of the COVID-19 pandemic on the psychosocial work environment and recovery among healthcare workers in a large university hospital in Sweden. J Public Health Res 2021;10. 10.4081/jphr.2021.2329. [Epub ahead of print: 14 07 2021]. PMC8715269 34278769

[26] Alexiou E , Steingrimsson S , Akerstrom M , et al . A survey of psychiatric healthcare workers' perception of working environment and possibility to recover before and after the first wave of COVID-19 in Sweden. Front Psychiatry 2021;12:770955. 10.3389/fpsyt.2021.770955 34912253 PMC8666504

[27] Demerouti E , Bakker AB , Nachreiner F , et al . The job demands-resources model of burnout. J Appl Psychol 2001;86:499–512. 10.1037/0021-9010.86.3.499 11419809

[28] Oude Hengel KM , In der Maur M , Bouwens L , et al . Reading Guide The Netherlands Working Conditions Survey - COVID-19: five waves (2019-2020-2021). TNO/CBS, 2022.

[29] Schaufeli WB , Van Dierendonck D . Handleiding van de Utrechtse burnout schaal (UBOS)[manual Utrecht burnout scale]. Lisse: Swets Test Services, 2000: 177–96.

[30] Karasek R , Brisson C , Kawakami N , et al . The job content questionnaire (JCQ): an instrument for internationally comparative assessments of psychosocial job characteristics. J Occup Health Psychol 1998;3:322–55. 10.1037/1076-8998.3.4.322 9805280

[31] Kristensen TS , Hannerz H , Høgh A , et al . The Copenhagen psychosocial Questionnaire—a tool for the assessment and improvement of the psychosocial work environment. Scand J Work Environ Health 2005;31:438–49. 10.5271/sjweh.948 16425585

[32] Rijksoverheid/Government . Coronavirus Dashboard: current situation in the Netherlands, 2022. Available: https://coronadashboard.government.nl/ [Accessed 05 Apr 2022].

[33] Cohen J . Statistical power analysis for the behavioural sciences. 2nd ed. New York: Psychology Press, 1988.

[34] Rimé B , Mesquita B , Boca S , et al . Beyond the emotional event: six studies on the social sharing of emotion. Cogn Emot 1991;5:435–65. 10.1080/02699939108411052

[35] Maunder RG , Heeney ND , Hunter JJ , et al . Trends in burnout and psychological distress in hospital staff over 12 months of the COVID-19 pandemic: a prospective longitudinal survey. J Occup Med Toxicol 2022;17:11. 10.1186/s12995-022-00352-4 35614505 PMC9132565

[36] Maunder RG , Heeney ND , Kiss A , et al . Psychological impact of the COVID-19 pandemic on hospital workers over time: relationship to occupational role, living with children and elders, and modifiable factors. Gen Hosp Psychiatry 2021;71:88–94. 10.1016/j.genhosppsych.2021.04.012 33971518 PMC8096521

[37] Harvey SB , Modini M , Joyce S , et al . Can work make you mentally ill? A systematic meta-review of work-related risk factors for common mental health problems. Occup Environ Med 2017;74:301–10. 10.1136/oemed-2016-104015 28108676

[38] Aronsson G , Theorell T , Grape T , et al . A systematic review including meta-analysis of work environment and burnout symptoms. BMC Public Health 2017;17:264. 10.1186/s12889-017-4153-7 28302088 PMC5356239

