# Supplementary material for: Impact of the COVID-19 pandemic on psychosocial work factors and emotional exhaustion among workers in the healthcare sector: a longitudinal study among 1915 Dutch workers
Source: Occup Environ Med. 2022 Nov 24;80(1):27–33. doi: 10.1136/oemed-2022-108478 (PMC9763172; doi:10.1136/oemed-2022-108478)
Supplement: Supplementary data [file oemed-2022-108478supp001.pdf]

Supplementary table 1. Univariate analyses for demographics and outcomes* between non-respondents compared to respondents for each specific measurement.

	Measurement 2 July 2020	Measurement 3 November 2020	Measurement 4 March 2021	Measurement 5 November 2021
	Non-respondents vs. respondents (n = 605/1,310)	Non-respondents vs. respondents (n = 673/1,242)	Non-respondents vs. respondents (n = 781/1,134)	Non-respondents vs. respondents (n = 913/1,002)
	OR (95% CI)	OR (95% CI)	OR (95% CI)	OR (95% CI)
Age				
19-34	1.11 (0.83, 1.48)	1.08 (0.77, 1.53)	1.27 (0.88, 1.84)	1.13 (0.76, 1.67)
35-44	Ref	Ref	Ref	Ref
45-54	0.89 (0.66, 1.20)	0.80 (0.56, 1.15)	0.73 (0.49, 1.07)	**0.58 (0.39, 0.86)**
55-65	0.76 (0.57, 1.01)	**0.59 (0.42, 0.83)**	**0.65 (0.46, 0.94)**	0.74 (0.51, 1.05)
Sex				
Male	0.87 (0.68, 1.13)	0.99 (0.74, 1.34)	1.09 (0.80, 1.49)	0.92 (0.66, 1.28)
Female	Ref	Ref	Ref	Ref
Household composition				
Single	1.16 (0.90, 1.49)	0.81 (0.60, 1.09)	0.93 (0.68, 1.28)	0.93 (0.68, 1.28)
Having a partner	1.15 (0.92, 1.44)	**0.61 (0.46, 0.80)**	0.91 (0.69, 1.21)	0.83 (0.62, 1.10)
Having a partner with child(ren)	Ref	Ref	Ref	Ref
Level of education				
Low	0.80 (0.52, 1.25)	0.94 (0.57, 1.56)	0.79 (0.46, 1.38)	1.15 (0.69, 1.93)
Middle	1.00 (0.82, 1.22)	1.08 (0.85, 1.37)	0.95 (0.73, 1.22)	0.97 (0.75, 1.26)
High	Ref	Ref	Ref	Ref
Working hours per week**	1.00 (0.99, 1.01)	-	0.99 (0.98, 1.01)	1.01 (0.99, 1.03)
Subgroup***				
Working with COVID-19 patients	-	**1.53 (1.02, 2.30)**	1.25 (0.87, 1.79)	0.88 (0.61, 1.27)
Working with other patients	-	**1.39 (1.07, 1.81)**	1.01 (0.77, 1.34)	0.79 (0.59, 1.05)
Not working with patients	-	Ref	Ref	Ref
Working conditions (mean, SD)				
Job autonomy (1-3)	0.87 (0.72, 1.06)	**0.76 (0.61, 0.96)**	0.93 (0.73, 1.18)	0.88 (0.69, 1.13)
Psychological job demands (1-4)	1.09 (0.93, 1.27)	1.01 (0.82, 1.24)	1.04 (0.84, 1.30)	1.13 (0.91, 1.42)
Emotional demands (1-4)	0.96 (0.80, 1.14)	0.99 (0.79, 1.24)	1.07 (0.85, 1.36)	1.07 (0.83, 1.38)
Social support from colleagues (1-4)	1.09 (0.91, 1.29)	1.02 (0.82, 1.26)	0.94 (0.75, 1.19)	0.82 (0.63, 1.05)
Social support from supervisor (1-4)	0.98 (0.85, 1.14)	**0.83 (0.69, 0.99)**	1.02 (0.84, 1.24)	1.00 (0.81, 1.23)
Emotional exhaustion (1-7) (mean, SD)	0.98 (0.90, 1.06)	1.00 (0.91, 1.10)	**1.13 (1.03, 1.25)**	1.08 (0.97, 1.19)

Bold indicates statistical significance ($p < 0.05$). *Outcomes and characteristics were retrieved from the previous measurement. **N/A for measurement 2. ***N/A for measurement 1.

Supplementary table 2. Hedges d effect sizes comparing healthcare workers working with (COVID-19) patients and healthcare workers not working with patients on changes in working conditions during the COVID-19 pandemic compared to baseline scores.

	Working with COVID-19 patients vs. not working with patients	Working with other patients vs. not working with patients
	Hedges d	Hedges d
Working conditions		
Job autonomy (1-3)	0.20	0.13
Psychological job demands (1-4)	0.10	0.05
Emotional demands (1-4)	0.16	0.10
Social support from colleagues (1-4)	0.10	0.04
Social support from supervisor (1-4)	0.08	0.06

Supplementary table 3. Characteristics of healthcare workers in the Netherlands (n = 1,915 at baseline) by period of measurement, stratified by sex.

	Measurement 1 November 2019 (n = 1,915)		Measurement 2 July 2020 (n = 1,310)		Measurement 3 November 2020 (n = 1,192)		Measurement 4 March 2021 (n = 1,046)		Measurement 5 November 2021 (n = 819)	
	Male (n = 352)	Female (n = 1563)	Male (n = 249)	Female (n = 1061)	Male (n = 222)	Female (n = 970)	Male (n = 185)	Female (n = 861)	Male (n = 148)	Female (n = 671)
Age										
19-34	73 (21%)	433 (28%)	48 (19%)	277 (26%)	40 (18%)	244 (25%)	23 (13%)	201 (23%)	19 (13%)	131 (20%)
35-44	61 (17%)	295 (19%)	43 (17%)	194 (18%)	37 (17%)	170 (18%)	33 (18%)	151 (18%)	20 (13%)	130 (19%)
45-54	74 (21%)	364 (23%)	56 (23%)	247 (23%)	50 (22%)	236 (24%)	45 (24%)	211 (24%)	41 (28%)	181 (27%)
55-65	144 (41%)	471 (30%)	102 (41%)	343 (33%)	95 (43%)	320 (33%)	84 (45%)	298 (35%)	68 (46%)	229 (34%)
Household composition										
Single	71 (20%)	359 (23%)	52 (21%)	236 (22%)	41 (19%)	234 (24%)	31 (17%)	218 (26%)	26 (18%)	159 (24%)
Having a partner	133 (38%)	505 (32%)	87 (35%)	341 (32%)	90 (40%)	328 (34%)	68 (37%)	287 (33%)	57 (38%)	218 (32%)
Having a partner with child(ren)	148 (42%)	699 (45%)	110 (44%)	484 (46%)	91 (41%)	408 (42%)	86 (46%)	356 (41%)	65 (44%)	294 (44%)
Level of education										
Low	17 (5%)	93 (6%)	12 (5%)	68 (6%)	13 (6%)	56 (6%)	10 (6%)	57 (7%)	8 (5%)	40 (6%)
Middle	108 (31%)	657 (42%)	78 (31%)	444 (42%)	68 (31%)	400 (41%)	56 (30%)	363 (42%)	46 (31%)	286 (43%)
High	227 (64%)	809 (52%)	159 (64%)	547 (52%)	141 (63%)	511 (53%)	119 (64%)	441 (51%)	94 (64%)	343 (51%)
Working hours per week (mean, SD)*	32.34 (9.66)	25.67 (8.36)	-	-	34.74 (6.64)	27.43 (6.82)	35.10 (5.97)	27.09 (6.72)	34.43 (7.27)	26.92 (7.07)
Subgroup										
Working with COVID-19 patients	-	-	25 (10%)	110 (10%)	35 (16%)	158 (16%)	28 (15%)	149 (17%)	30 (20%)	139 (21%)
Working with other patients	-	-	93 (37%)	556 (52%)	72 (32%)	468 (48%)	57 (31%)	413 (48%)	45 (30%)	328 (49%)
Not working with patients	-	-	120 (48%)	347 (33%)	108 (49%)	310 (32%)	90 (49%)	259 (30%)	68 (46%)	174 (26%)
Working conditions (mean, SD)										
Job autonomy (1-3)	2.51 (0.48)	2.37 (0.49)	2.48 (0.50)	2.29 (0.52)	2.52 (0.49)	2.29 (0.52)	2.51 (0.51)	2.29 (0.52)	2.51 (0.51)	2.29 (0.52)
Psychological job demands (1-4)	2.33 (0.61)	2.47 (0.63)	2.25 (0.53)	2.36 (0.58)	2.27 (0.54)	2.37 (0.59)	2.25 (0.55)	2.32 (0.58)	2.32 (0.61)	2.41 (0.65)
Emotional demands (1-4)	2.15 (0.59)	2.22 (0.54)	2.15 (0.58)	2.21 (0.51)	2.09 (0.58)	2.20 (0.52)	2.04 (0.56)	2.16 (0.50)	2.11 (0.65)	2.19 (0.52)
Social support from colleagues (1-4)	3.39 (0.55)	3.44 (0.57)	3.40 (0.55)	3.49 (0.55)	3.44 (0.50)	3.49 (0.55)	3.43 (0.51)	3.49 (0.52)	3.45 (0.50)	3.49 (0.55)
Social support from supervisor (1-4)	3.05 (0.65)	3.01 (0.66)	3.08 (0.64)	3.08 (0.68)	3.12 (0.64)	3.07 (0.68)	3.22 (0.65)	3.09 (0.64)	3.12 (0.62)	3.06 (0.68)
Emotional exhaustion (1-7) (mean, SD)	2.27 (1.24)	2.36 (1.25)	2.27 (1.17)	2.46 (1.26)	2.25 (1.21)	2.44 (1.25)	2.15 (1.16)	2.40 (1.26)	2.21 (1.22)	2.58 (1.39)

*N/A for measurement 2.

Supplementary table 4. Linear mixed model estimates for differences across healthcare workers in psychosocial working conditions and emotional exhaustion during the COVID-19 period (measurement 2-5), without correction for hospital pressure and baseline scores (measurement 1)

	Job autonomy (1-3)	Psychological job demands (1-4)	Emotional demands (1-4)	Social support from colleagues (1-4)	Social support from supervisor (1-4)	Emotional exhaustion (1-7)
	β (95% CI)	β (95% CI)	β (95% CI)	β (95% CI)	β (95% CI)	β (95% CI)
**Subgroup**						
Working with COVID-19 patients	**-0.23 (-0.27, -0.18)**	**0.15 (0.10, 0.20)**	**0.19 (0.14, 0.24)**	**0.07 (0.01, 0.12)**	**-0.11 (-0.18, -0.04)**	0.11 (-0.003, 0.22)
Working with other patients	**-0.15 (-0.18, -0.12)**	**0.08 (0.04, 0.12)**	**0.15 (0.11, 0.18)**	0.03 (-0.01, 0.07)	**-0.10 (-0.16, -0.05)**	0.06 (-0.02, 0.15)
Not working with patients	Ref	Ref	Ref	Ref	Ref	Ref

Bold indicates statistical significance (p < 0.05). Analyses were corrected for sex, age, household composition, level of education and working hours.

Supplementary table 5. Linear fixed effects regression model estimates for the effect of change between working with other patients and working with COVID-19 patients on change in psychosocial working conditions and emotional exhaustion during the COVID-19 period (measurement 2-5), with and without adjusting for hospital pressure.

	Job autonomy (1-3)		Psychological job demands (1-4)		Emotional demands (1-4)		Social support from colleagues (1-4)		Social support from supervisor (1-4)		Emotional exhaustion (1-7)	
	β (95% CI)		β (95% CI)		β (95% CI)		β (95% CI)		β (95% CI)		β (95% CI)	
	No adjustment	Adj. for hospital pressure	No adjustment	Adj. for hospital pressure	No adjustment	Adj. for hospital pressure	No adjustment	Adj. for hospital pressure	No adjustment	Adj. for hospital pressure	No adjustment	Adj. for hospital pressure
**Subgroup**	-0.02 (-0.05, 0.01)	-0.02 (-0.05, 0.02)	0.01 (-0.03, 0.05)	0.01 (-0.03, 0.05)	0.02 (-0.02, 0.06)	0.03 (-0.01, 0.07)	**0.05 (0.01, 0.10)**	**0.06 (0.01, 0.10)**	-0.01 (-0.07, 0.05)	-0.02 (-0.08, 0.04)	**0.11 (0.03, 0.20)**	**0.11 (0.02, 0.19)**

Bold indicates statistical significance (p < 0.05).
